# Supplementary material for: Conformational flexibility in neutralization of SARS-CoV-2 by naturally elicited anti-SARS-CoV-2 antibodies
Source: Commun Biol. 2022 Aug 5;5:789. doi: 10.1038/s42003-022-03739-5 (PMC9355996; doi:10.1038/s42003-022-03739-5)
Supplement: Supplementary file 3 — Reporting Summary [file 42003_2022_3739_MOESM3_ESM.pdf]

nature research

Corresponding author(s): Natalia Freund

Last updated by author(s): May 24, 2022

Reporting Summary

Nature Research wishes to improve the reproducibility of the work that we publish. This form provides structure for consistency and transparency in reporting. For further information on Nature Research policies, see our Editorial Policies and the Editorial Policy Checklist.

Statistics

For all statistical analyses, confirm that the following items are present in the figure legend, table legend, main text, or Methods section.

n/a	Confirmed	
☐	☒	The exact sample size (*n*) for each experimental group/condition, given as a discrete number and unit of measurement
☒	☐	A statement on whether measurements were taken from distinct samples or whether the same sample was measured repeatedly
☐	☒	The statistical test(s) used AND whether they are one- or two-sided<br>*Only common tests should be described solely by name; describe more complex techniques in the Methods section.*
☒	☐	A description of all covariates tested
☐	☒	A description of any assumptions or corrections, such as tests of normality and adjustment for multiple comparisons
☐	☒	A full description of the statistical parameters including central tendency (e.g. means) or other basic estimates (e.g. regression coefficient) AND variation (e.g. standard deviation) or associated estimates of uncertainty (e.g. confidence intervals)
☒	☐	For null hypothesis testing, the test statistic (e.g. *F*, *t*, *r*) with confidence intervals, effect sizes, degrees of freedom and *P* value noted<br>*Give P values as exact values whenever suitable.*
☒	☐	For Bayesian analysis, information on the choice of priors and Markov chain Monte Carlo settings
☒	☐	For hierarchical and complex designs, identification of the appropriate level for tests and full reporting of outcomes
☒	☐	Estimates of effect sizes (e.g. Cohen's *d*, Pearson's *r*), indicating how they were calculated

Our web collection on statistics for biologists contains articles on many of the points above.

Software and code

Policy information about availability of computer code

Data collection	Crystal structure: Data were processed and scaled with HKL2000. The structure was determined using PHASER. Manual building and adjustments of the were performed in COOT. The structure of RBD-Fab2303 was refined by using PHENIX. Structural analysis of antibody–antigen contacts were assessed through CCP4i. All structural representations were prepared through UCSF Chimera and ChimeraX.<br>CryoEM: Motion Cor2, CTF parameters of the micrographs were determined by using the program Gctf, particles were boxed by using Gautomatch, and were then subjected to 2D classification using RELION
Data analysis	GraphPad Prism versions 8 and 9, FlowJo 10.7.1, IncuCyte ZOOM, default IncuCyte software

For manuscripts utilizing custom algorithms or software that are central to the research but not yet described in published literature, software must be made available to editors and reviewers. We strongly encourage code deposition in a community repository (e.g. GitHub). See the Nature Research guidelines for submitting code & software for further information.

Data

Policy information about availability of data

All manuscripts must include a data availability statement. This statement should provide the following information, where applicable:

- Accession codes, unique identifiers, or web links for publicly available datasets
- A list of figures that have associated raw data
- A description of any restrictions on data availability

Data and materials availability
The atomic coordinates and EM maps have been deposited into the Protein Data Bank (http://www.pdb.org) and the EM Data Bank, respectively: Fab2303-RBD complex (PDB: 7WBZ), Fab2303-S complex (EMD: 32411), Fab2212 (PDB: 7WC0), mAb2212-S complex in conformation 1 (EMD: 32416), conformation 2 (EMD: 32417), conformation 3 (EMD: 32418), conformation 4 (EMD: 32421, PDB: 7WCD), conformation 5 with head-to-head spikes (EMD: 32420) and conformation 5 with

single spike (EMD: 32419).

Field-specific reporting

Please select the one below that is the best fit for your research. If you are not sure, read the appropriate sections before making your selection.

☒ Life sciences ☐ Behavioural & social sciences ☐ Ecological, evolutionary & environmental sciences

For a reference copy of the document with all sections, see nature.com/documents/nr-reporting-summary-flat.pdf

Life sciences study design

All studies must disclose on these points even when the disclosure is negative.

Sample size	Each ELISA experiment was repeated at least 3 times. For antibody inhibition by flow cytometry, 3x10^6 cells were used as a starting point.<br>For pseudo virus experiments three replicates were analyzed, the experiments were preformed twice.<br>For authentic virus neutralization the experiments were preformed 2-6 times (at .least twice)
Data exclusions	No data was excluded.
Replication	All experiments were replicated successfully.
Randomization	Not applicable to this study.
Blinding	Not applicable to this study.

Reporting for specific materials, systems and methods

We require information from authors about some types of materials, experimental systems and methods used in many studies. Here, indicate whether each material, system or method listed is relevant to your study. If you are not sure if a list item applies to your research, read the appropriate section before selecting a response.

Materials & experimental systems

n/a	Involved in the study	
☐	☒	Antibodies
☐	☒	Eukaryotic cell lines
☒	☐	Palaeontology and archaeology
☒	☐	Animals and other organisms
☒	☐	Human research participants
☒	☐	Clinical data
☒	☐	Dual use research of concern

Methods

n/a	Involved in the study	
☒	☐	ChIP-seq
☐	☒	Flow cytometry
☒	☐	MRI-based neuroimaging

Antibodies

Antibodies used	All anti-SARS-CoV-2 TAU antibodies were isolated and produced in our lab. DNA of mGO53 mAb was provided by the laboratory of Dr. Michal Nussenzweig, Rockefeller University, and produced in our lab.<br>Goat Anti-Human IgG HRP (Jackson ImmunoResearch, Catalog: 109-035-088, Dilution: 1:5000).<br>Rabbit anti-SARS-CoV-2 (COVID-19) nucleocapsid antibody (GeneTex, Catalog: GTX135357).<br>Goat anti-Rabbit IgG (H+L) Highly Cross-Adsorbed Secondary Antibody, Alexa Fluor™ 594 (A-11037).
Validation	All TAU mAbs were validated in this study and in Mor M et al, PLOS Pathogens, 2021. mGO53 was validated in Wardemann H et al, Science, 2003. The validation of commercially available antibodies used in this study was described in technical data sheets provided by the manufacturers and/or on their websites.

Eukaryotic cell lines

Policy information about cell lines

Cell line source(s)	Expi293F Cells - Thermofisher Scientific.<br>VeroE6 - Sekisui XenoTech<br>Calu-3 ATCC<br>Sf9 cells<br>Hi5 cells
Authentication	The Cell lines are regularly used and checked for growth rate. The cell stock is replaced after 20 passages.
Mycoplasma contamination	Cells were tested 2-3 years ago for mycoplasma. Since then new stocks have been used, with no contamination concern.
Commonly misidentified lines<br>(See ICLAC register)	N/A

Flow Cytometry

Plots

Confirm that:

☒ The axis labels state the marker and fluorochrome used (e.g. CD4-FITC).

☐ The axis scales are clearly visible. Include numbers along axes only for bottom left plot of group (a 'group' is an analysis of identical markers).

☒ All plots are contour plots with outliers or pseudocolor plots.

☒ A numerical value for number of cells or percentage (with statistics) is provided.

Methodology

Sample preparation	All the methodologies is indicated in the manuscript
Instrument	CytoFLEX S Flow Cytometer - Beckman Coulter. all other used instruments are indicated in the manuscript
Software	Data was collected using CytExpert software and analyzed using FlowJo. indicated in the manuscript
Cell population abundance	No post-sort cell fraction were used for further analyze.
Gating strategy	Gating strategy is described in Extended Data Fig. 2a.

☒ Tick this box to confirm that a figure exemplifying the gating strategy is provided in the Supplementary Information.
