# Supplementary material for: Conformational flexibility in neutralization of SARS-CoV-2 by naturally elicited anti-SARS-CoV-2 antibodies
Source: Commun Biol. 2022 Aug 5;5:789. doi: 10.1038/s42003-022-03739-5 (PMC9355996; doi:10.1038/s42003-022-03739-5)
Supplement: Supplementary file 2 — Supplementary Information [file 42003_2022_3739_MOESM2_ESM.pdf]

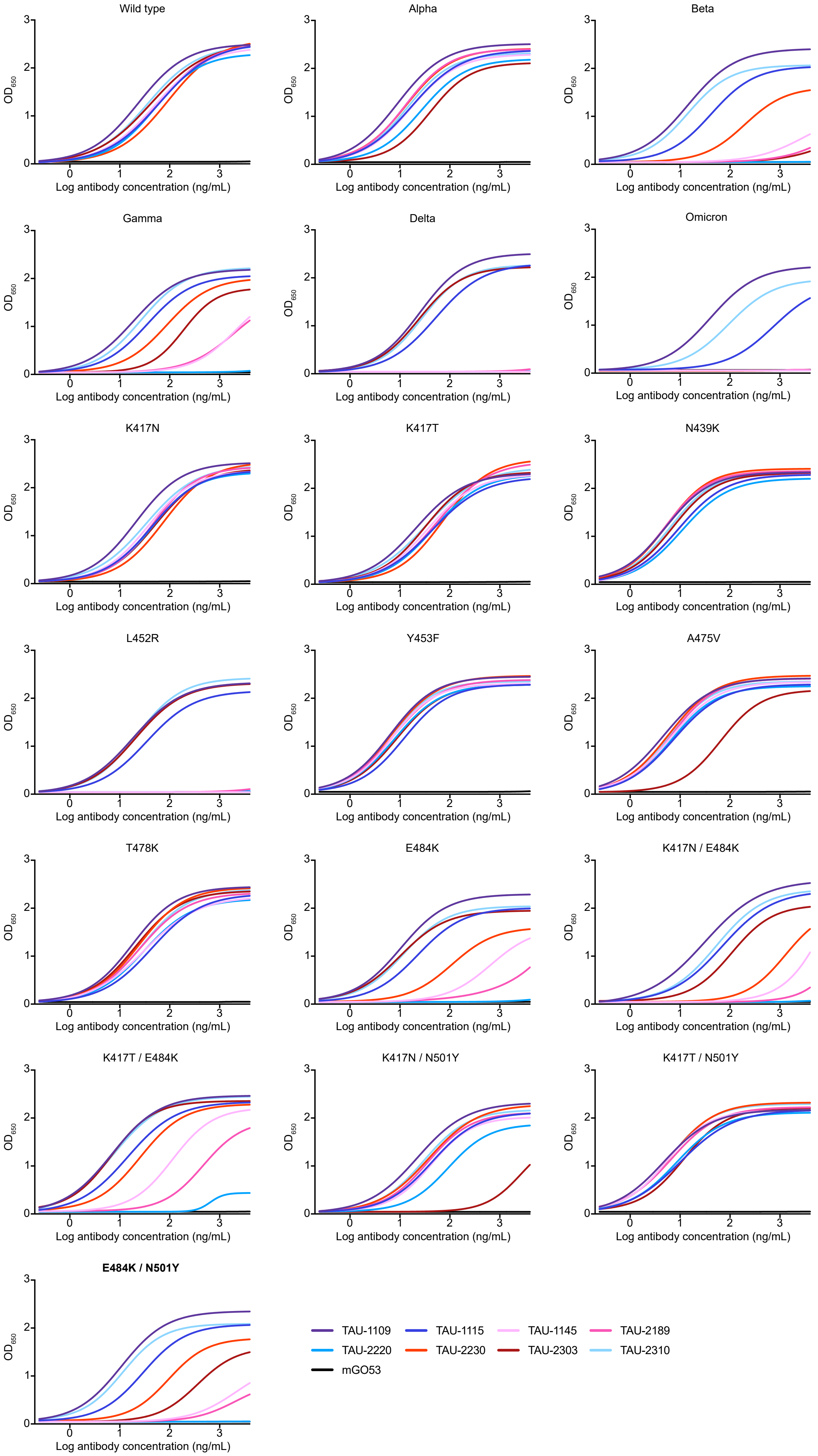

Supplementary Figure 1: Antibody binding to wild type SARS-CoV-2 and VOCs RBDs by ELISA. Representative ELISA curves for the eight RBD-binding mAbs against (indicated on the bottom) to wild type RBD and RBDs harboring single, double or triple mutations corresponding to common SARS-CoV-2 VOCs. The y axis shows OD650 values, and the x axis shows Log [mAb concentration], starting with 10 µg/mL, and with 8 consecutive 4-fold dilutions. mGO53 was used as an isotype control antibody.

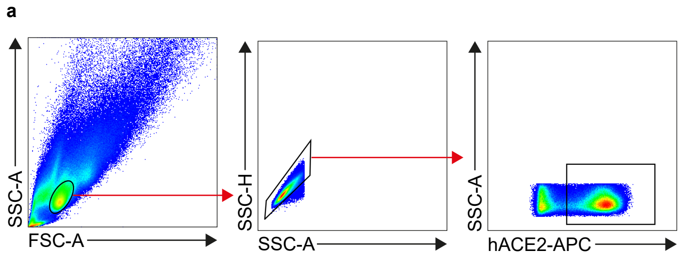

Supplementary Figure 2: Inhibition of hACE2 binding to cell expressed spike by antibodies as measured by flow cytometry. **a**, Gating strategy used to measure antibody inhibition of spike:ACE2 binding. b-e, Representative flow cytometry plots of positive hACE2 cells expressing wild type (**b**), Alpha (**c**), Beta (**d**) and Delta (**e**) spikes in the presence of mAbs. mGO53 was used as an isotype control. Unlabeled ("cold" hACE2) was used as positive control for inhibition.

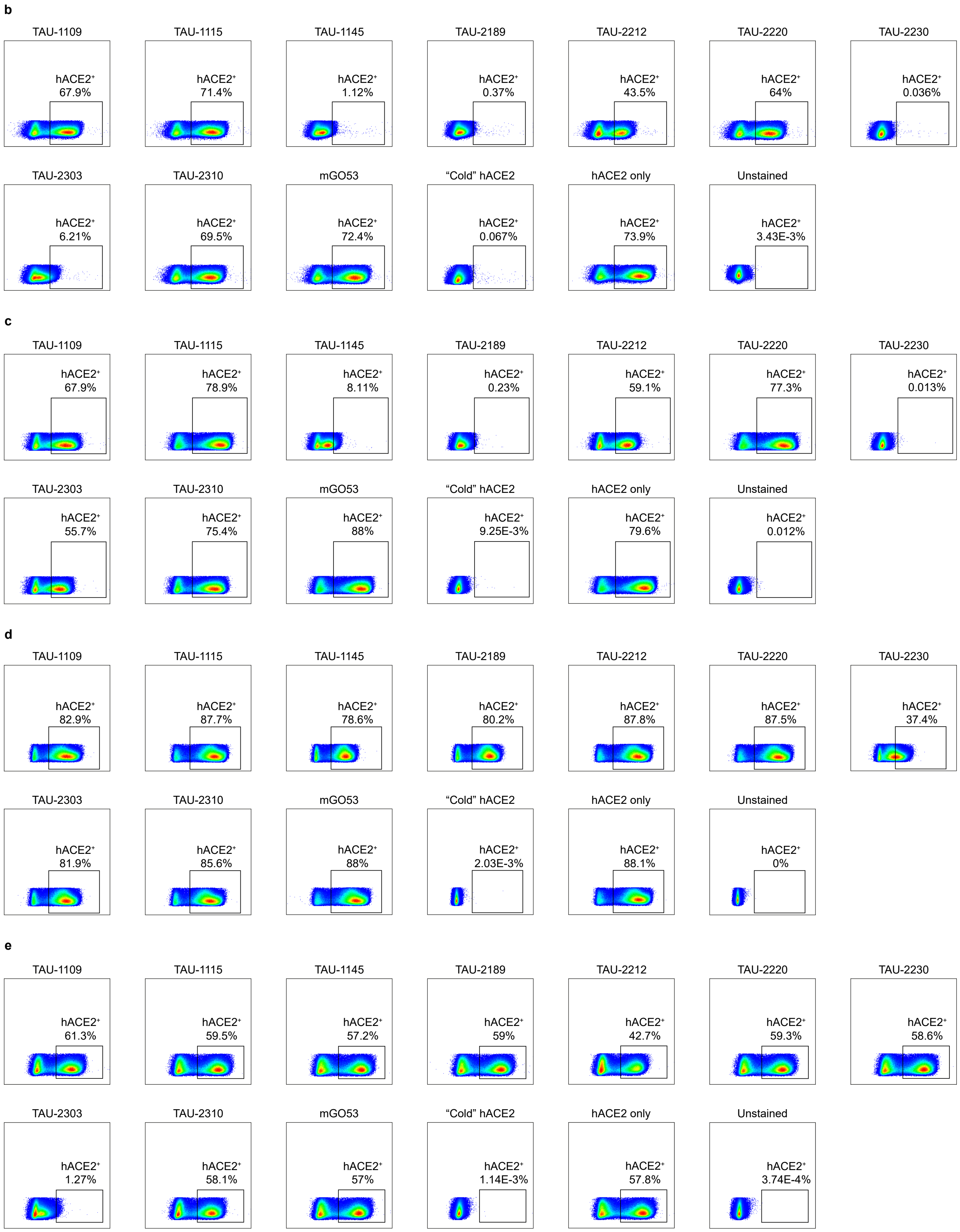

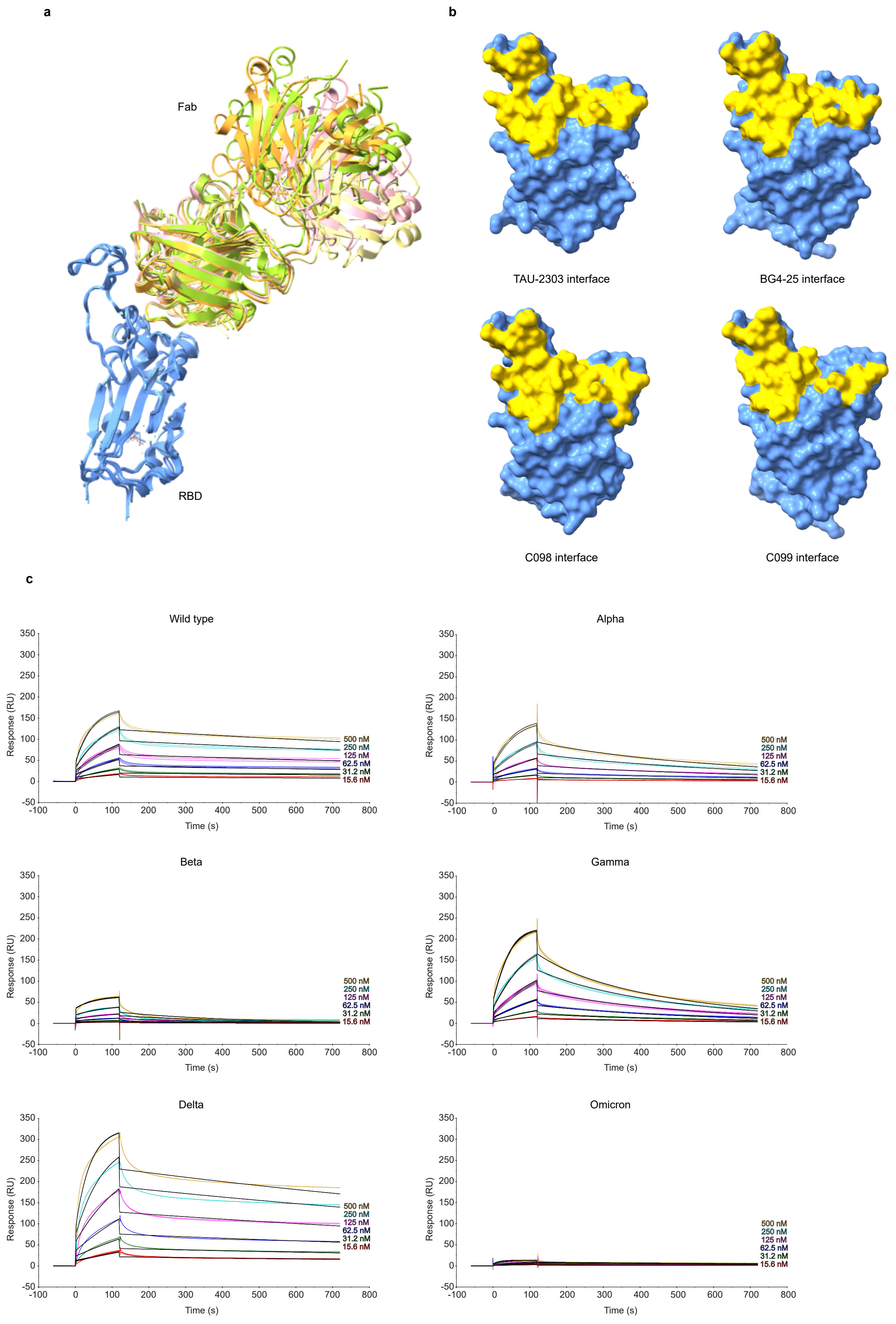

d

RBD	Wild type	Alpha	Beta	Gamma	Delta	Omicron
Rmax (RU)	133.8	111.8	<50	187.7	245.8	<50
KD (M)	9.85E-09	4.22E-08	-	5.39E-08	9.76E-09	-
U-Value	4	3	-	2	3	-

Supplementary Figure 3: Epitope of TAU-2303. **a**, Comparison between the epitope of TAU-2303 and other Class 1 mAbs. Ribbon diagrams showing the comparisons of binding directions to RBD among Fab2303 (yellow), BG4-25[31] (green), C098[32] (pink) and C099[32] (orange). The Fab-RBD complexes are superimposed with the RBD (blue). **b**, Comparisons of contact interfaces on RBD. The surface of RBDs are colored blue and the binding epitopes are in yellow. **c**, SPR sensograms showing binding of injected SARS-CoV-2 RBDs (wild type and VOC) at six different concentrations (15.6 nM, 31.25 nM, 62.5 nM, 125 nM, 250 nM and 500 nM) to immobilized anti-SARS-CoV-2 TAU-2303 mAb (0.5 µg/ml). The wild type histogram is taken from our privous study[9]. d, SPR assays were performed on a Biacore T200 instrument at 25°C. Two to four replicates were performed for each mAb and all samples were diluted in HBS-EP buffer (0.01 M HEPES, 0.15 M NaCl, 0.003 M EDTA, 0.05% Tween 20, pH 7.4). Sensograms were fitted to 1:1 binding model using non-linear regression in the biaevaluation software. KD was calculated using the ratio of the kinetic constants KD = Kd/Ka. **d**, Table showing Rmax, KD, and U-value values.

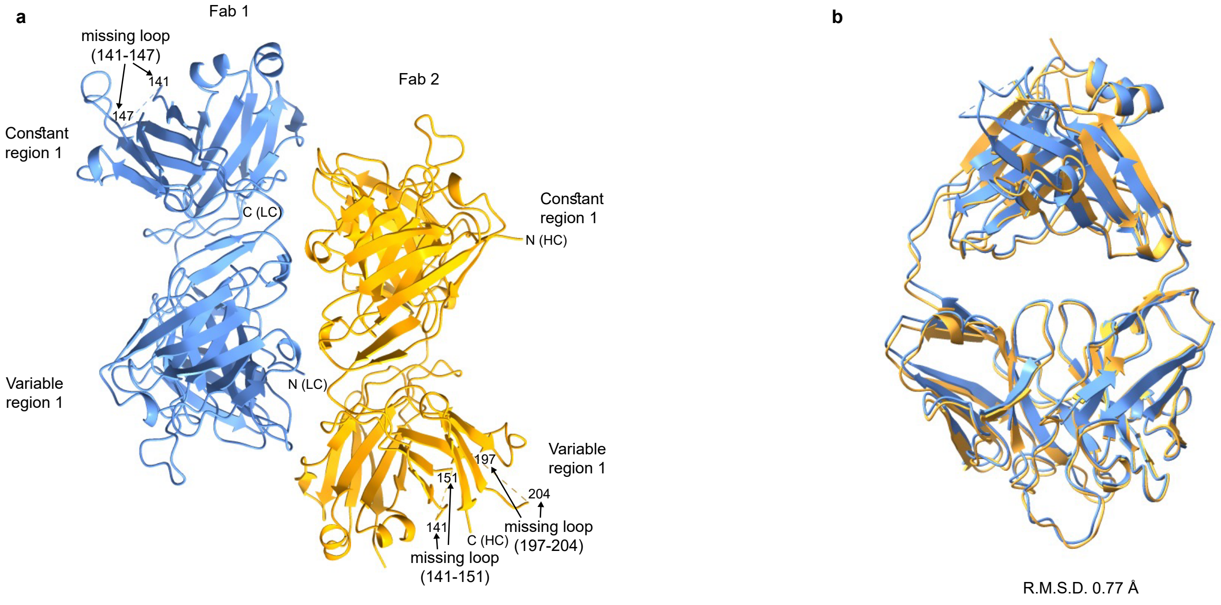

Supplementary Figure 4: Crystal structure of Fab2212. **a**, Ribbon diagrams showing two Fab2212 molecules in the asymmetric unit of the crystal. **b**, Superimposition of the two Fab2212 molecules in the asymmetric unit of the crystal.

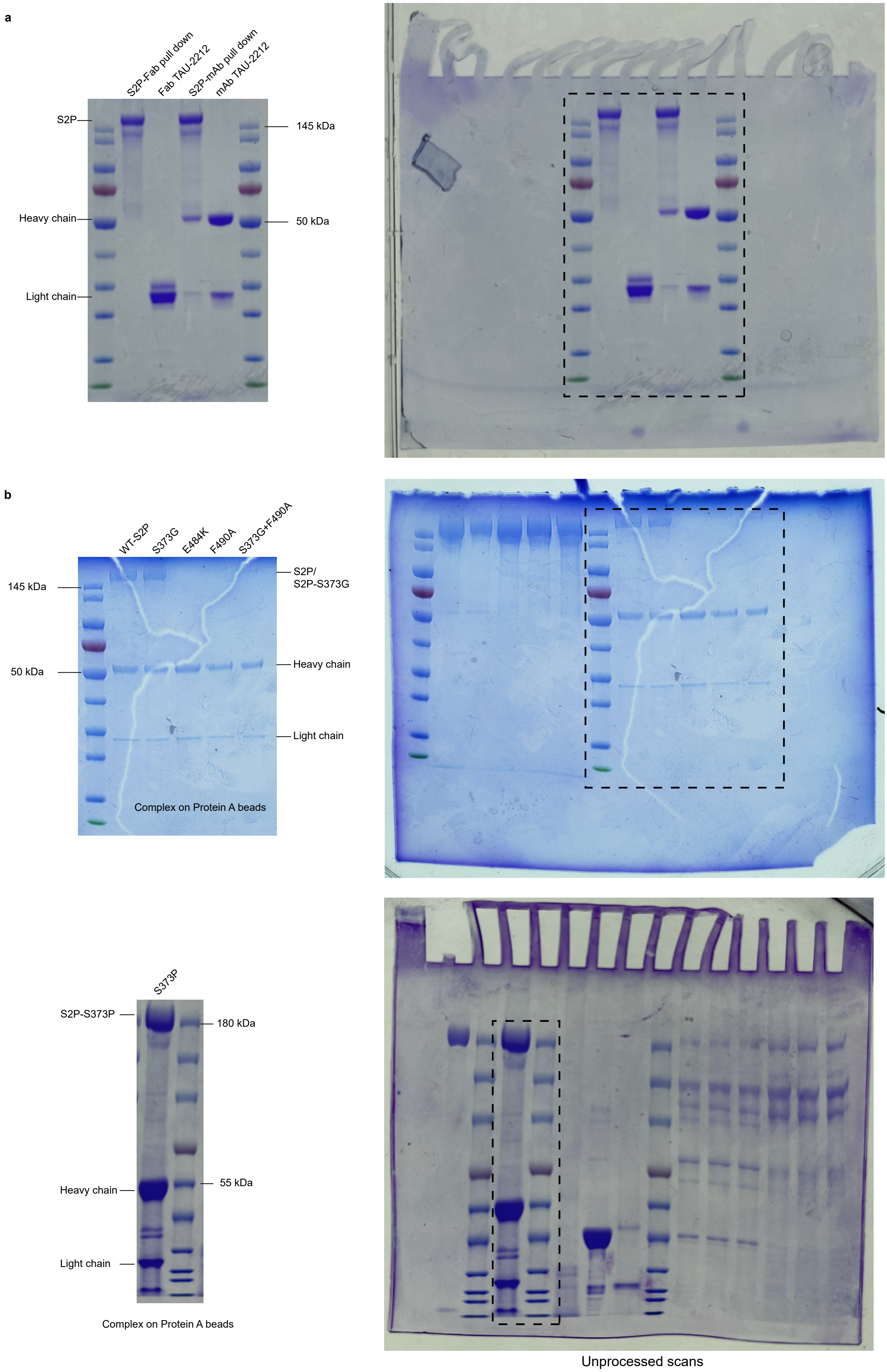

Supplementary Figure 5: Binding of mAb TAU-2212 or Fab TAU-2212 to spike trimer. **a**, SDS-PAGE gel analysis showing the pull-down of mAb TAU-2212 or Fab TAU-2221 by S2P. **b**, SDS-PAGE gel analysis showing the pull-downs of S2P or S2P mutants by mAb TAU-2212.

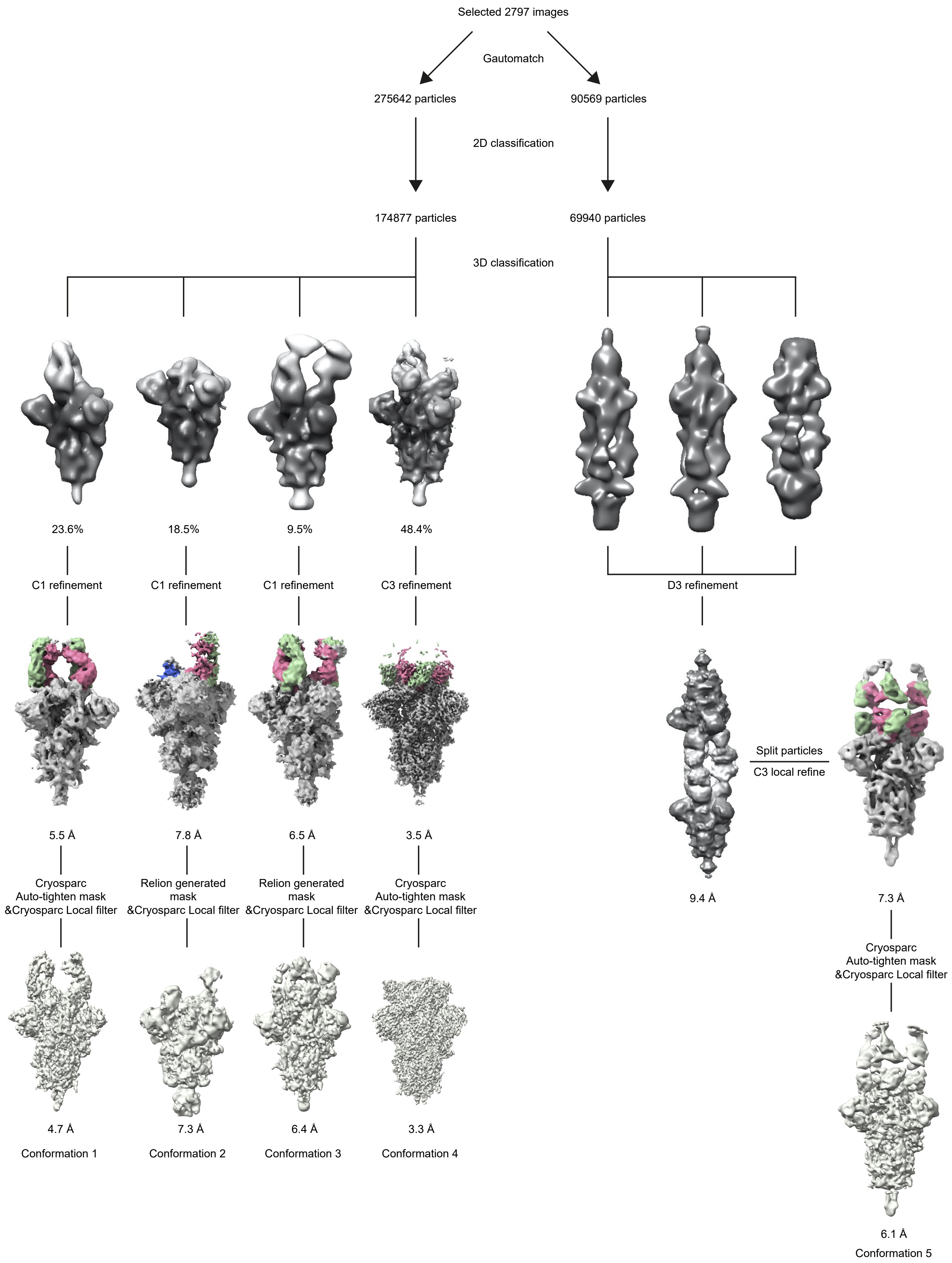

Supplementary Figure 6: Cryo-EM data processing workflow for the complex of TAU-2212 and spike trimer. Particles were picked by Gautomatch v0.56. Particle extraction, 2D classification, 3D classification and 3D refinements were performed using Relion 3.0.8. The refined half maps were applied to cryoSPARC v3.3.2 for postprocessing with local filter and masks either automatically generated by cryoSPARC or by Relion.

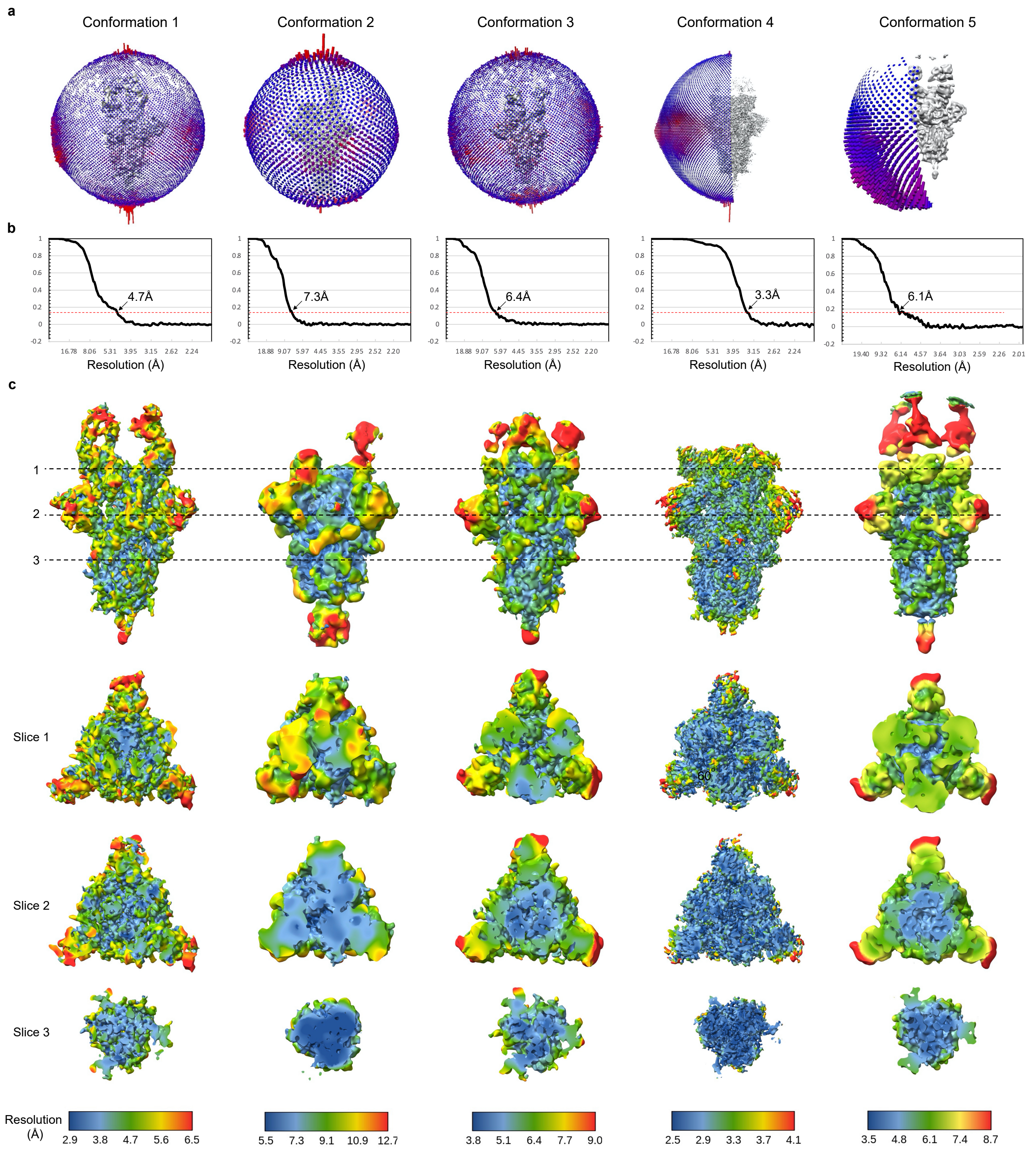

Supplementary Figure 7: Orientation distributions, FSC curves and local resolution maps of the reconstructions of the five TAU-2212-S2P conformations. a. The orientation distributions of particles used for final refinements of each reconstruction. b. Fourier shell coefficient curves of the reconstructions. The FSC threshold is 0.143 and xml files are from cryoSPARC FSC validation jobs. c. Local resolution maps were calculated with half maps by using cryoSPARC [Kucukelbir A, Sigworth FJ, Tagare HD. Quantifying the local resolution of cryo-EM density maps. Nat Methods. 2014 Jan;11(1):63-5. doi: 10.1038/nmeth.2727. Epub 2013 Nov 10. PMID: 24213166; PMCID: PMC3903095.].

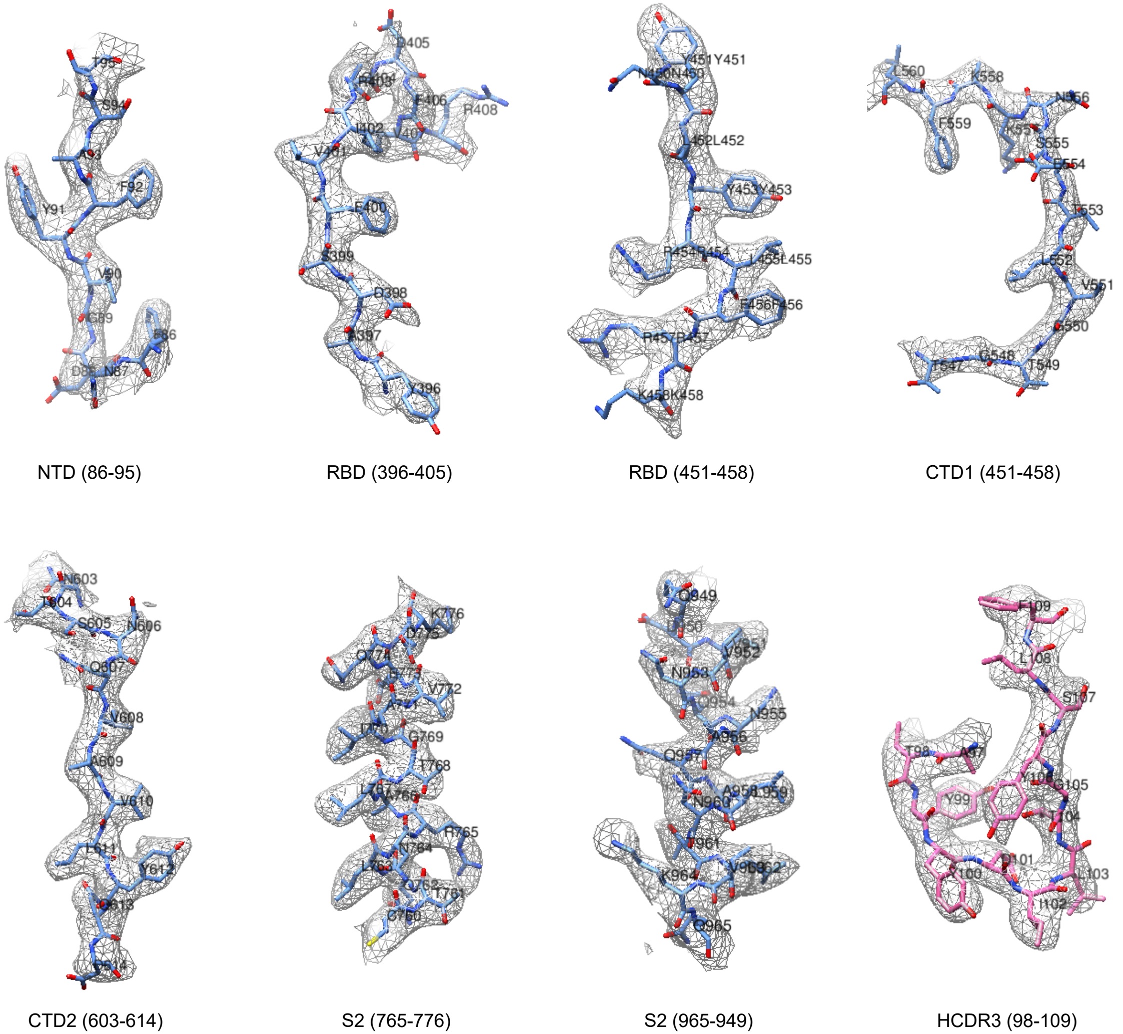

Supplementary Figure 8: Representative densities of the spike trimer with three bound TAU-2212 mAbs (conformation 4). The structures of the spike trimer are shown in sticks with C, O and N atoms colored light blue, red, and dark blue, respectively. The structure of the bound TAU-2212 is shown in sticks with C, O, and N atoms colored pink, red, and dark blue, respectively. The density maps are drawn at a contouring level of 0.016 e-/ Å3.

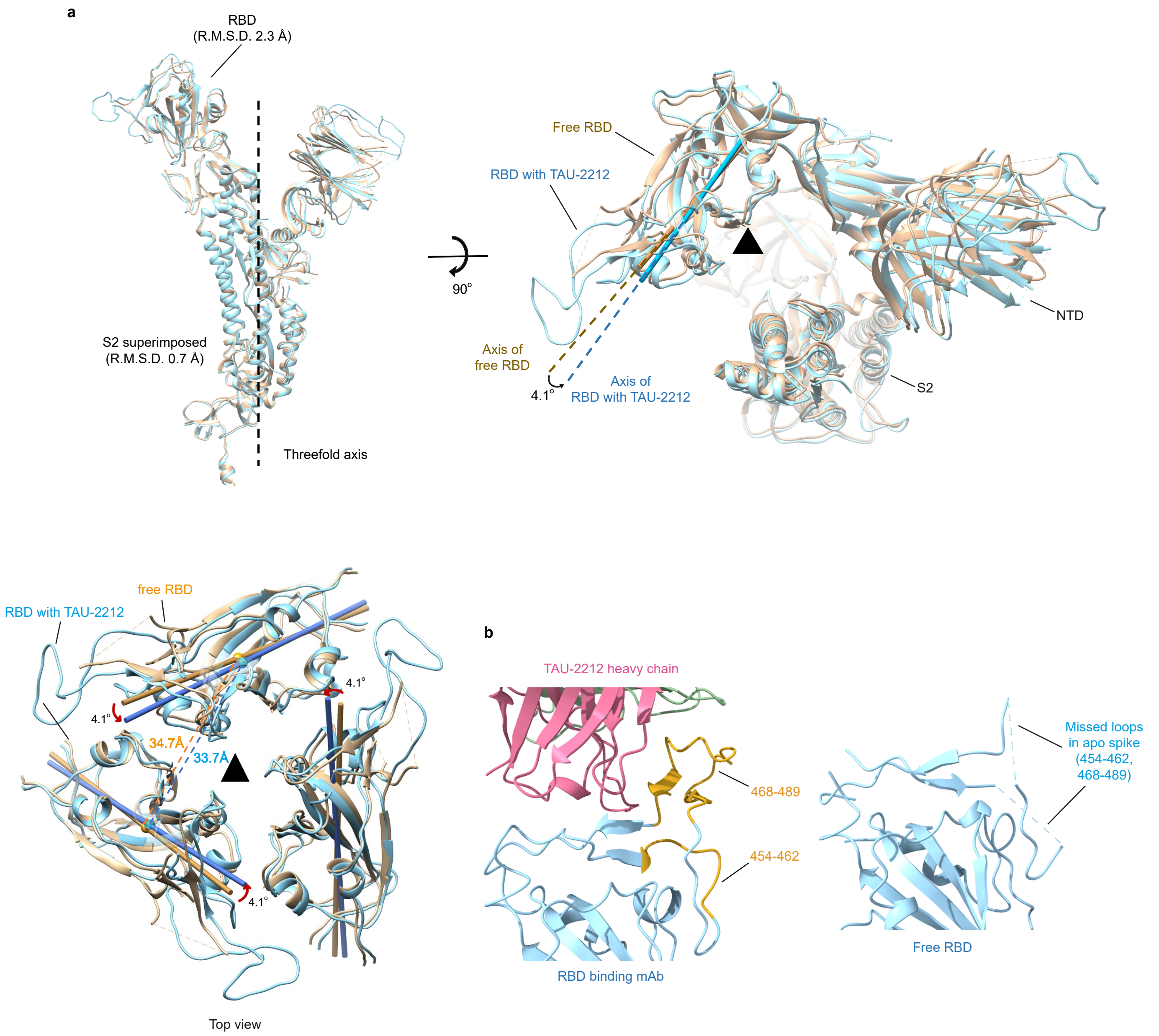

Supplementary Figure 9: Structural comparisons of TAU-2212 bound and free spike trimers. **a**, Top panel: ribbon diagrams showing one protomer of the TAU-2212 bound spike trimer superimposed on one protomer of the TAU-2212 free spike trimer. Superimpositions of the structures were performed with the S2 portion only. Bottom panel: ribbon diagrams and the long axes of the aligned RBDs showing the TAU-2212 binding induced conformational changes. **b**, Ribbon diagrams showing that loops 454-462 and 468-489 become ordered upon TAU-2212 binding.

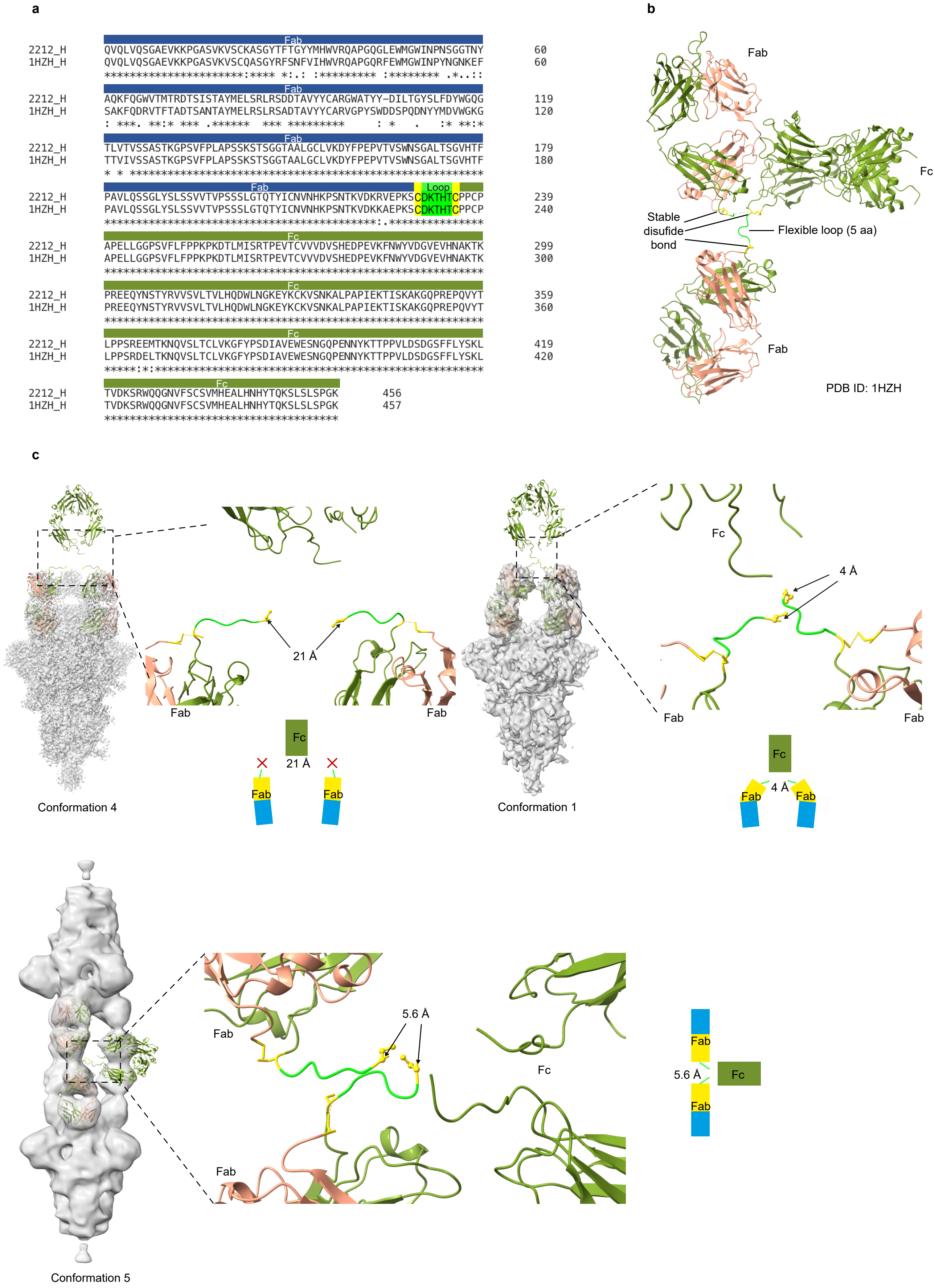

Supplementary Figure 10: Structural modeling of the spike trimer bound by full length TAU-2212. a, Sequence alignment between the TAU-2212 heavy chain and the human IgG B12 heavy chain [Saphire EO, Parren PW, Pantophlet R, Zwick MB, Morris GM, Rudd PM, Dwek RA, Stanfield RL, Burton DR, Wilson IA. Crystal structure of a neutralizing human IGG against HIV-1: a template for vaccine design. Science. 2001 Aug 10;293(5532):1155-9. doi: 10.1126/science.1061692. PMID: 11498595.]. The flexible loop "DKTHT" that connects the Fc and Fab of the antibody is highlighted in lime and the cysteines that form a disulfide bond between the loops are highlighted in yellow. b. Ribbon diagram of the crystal structure of human IgG B12, PDB ID:1HZH. Heavy chains are in green and light chains are in salmon. The disulfide bonds are in yellow and the flexible loops are colored lime. c. B12 Fab fitted in the reconstructed maps of conformations 1, 4, 5. The constant and variable regions of the Fab were fitted separately as rigid bodies. Zoom in ribbon diagrams exhibit the distances between two fitted Fabs. The distance between the Calpha atoms of the two C254s was measured and indicated. Schematic diagram showing the 3 scenarios. The constant region is shown in yellow and the variable region is in blue.

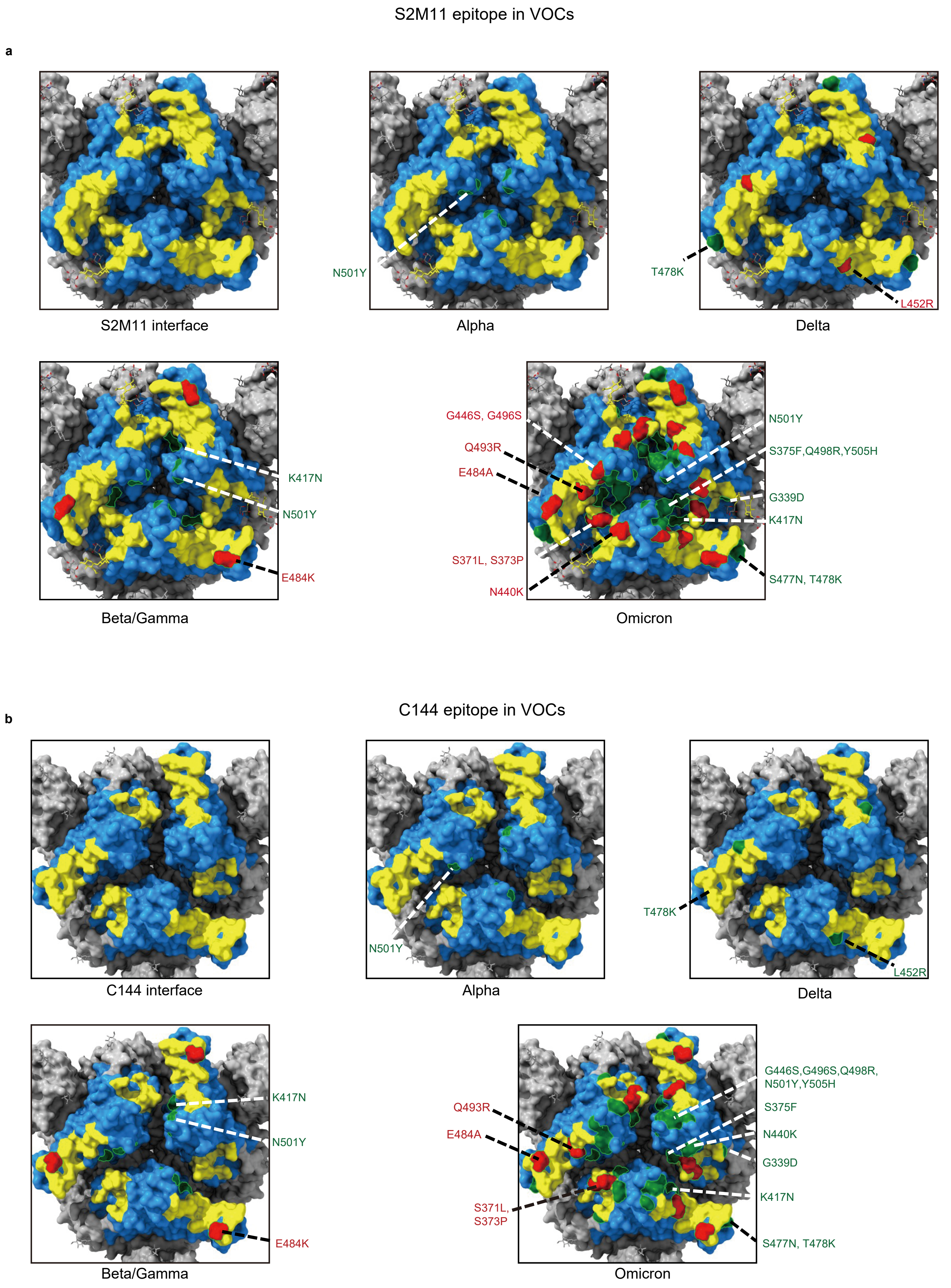

Supplementary Figure 11: Epitopes of antibodies similar to TAU-2212. Contact epitopes of S2M11 (**a**) and C144 (**b**) are shown in yellow. Key mutation sites within or outside the binding epitope of TAU-2303 are colored red and green, respectively.

		Variant of concern				
	Mutation	Alpha	Beta	Gamma	Delta	Omicron
	L18F			■		
	T20N			■		
	P26S			■		
	A67V					■
	Δ69-70	■				■
	D80A		■			
	T95I					■
	P138Y			■		
	G142D					■
	Δ143					■
	Δ144-145	■				■
	R190S			■		
	N211I					■
	Δ212					■
	214EPEins					■
	D215G		■			
	Δ242-244		■			
	G339D					■
	S371L					■
	S373P					■
	S375F					■
	K417N		■			■
	K417T			■		
	N440K					■
	G446S					■
**RBD**	L452R				■	
	S477N					■
	T478K				■	■
	E484A					■
	E484K		■	■		
	Q493R					■
	G498R					■
	N501Y	■	■	■		■
	Y505H					■
	T547K					■
	A570D	■				
	D614G	■	■	■	■	■
	H655Y			■		■
	N679K			■		■
	P681H	■				■
	P681R				■	
	A701V		■			
	T716I	■				
	N764K					■
	D796Y					■
	N856K					■
	Q954H					■
	N969K					■
	L981F					■
	S982A	■				
	T1027I			■		
	D1118H	■				
	V1176F			■		

Supplementary Table 1: Spike and RBD mutations of the Alpha, Beta, Gamma, Delta and Omicron VOCs.

Van der Waals contacts[a]		Direct hydrogen bonds[b]		
RBD	Fab 2303	RBD	Fab 2303	Distance (Å)
ARG^{403}	ASN^{L92}(4), ASN^{L93}(1)	ARG^{403}[NH1]	ASN^{L92}[O]	3.0
THR^{415}	SER^{H56}(2), TYR^{H58}(7)	THR^{415}[OG1]	TYR^{H58}[OH]	2.7
GLY^{416}	TYR^{H52}(3), TYR^{H58}(2)			
***LYS^{417}**	TYR^{H33}(2), TYR^{H52}(4), ASN^{L92}(1)	LYS^{417}[N]	TYR^{H52}[OH]	3.6
		LYS^{417}[NZ]	TYR^{H52}[OH]	3.6
ASP^{420}	TYR^{H52}(2), SER^{H56}(4)	ASP^{420}[OD2]	SER^{H56}[OG]	2.5
TYR^{421}	TYR^{H33}(2), TYR^{H52}(5), SER^{H53}(4), GLY^{H54}(2)	TYR^{421}[OH]	SER^{H53}[N]	3.3
		TYR^{421}[OH]	GLY^{H54}[N]	3.1
**TYR^{453}**	VAL^{H101}(1), ASN^{L92}(2)	TYR^{453}[OH]	ASN^{L92}[ND2]	2.9
**LEU^{455}**	TYR^{H33}(5), ALA^{H100}(2), VAL^{H101}(1)	LEU^{455}[O]	TYR^{H33}[OH]	2.7
ARG^{457}	SER^{H53}(2)			
LYS^{458}	ARG^{H30}(11), SER^{H53}(3), GLY^{H54}(2)	LYS^{458}[O]	ARG^{H30}[NH2]	2.9
		SER^{459}[OG]	ARG^{H30}[NH1]	3.3
ASN^{460}	GLY^{H54}(5)			
TYR^{473}	SER^{H31}(6), SER^{H53}(2)	TYR^{473}[OH]	SER^{H31}[O]	2.6
		TYR^{473}[OH]	SER^{H53}[OG]	3.4
GLN^{474}	SER^{H31}(1)			
**ALA^{475}**	PHE^{H27}(2), THR^{H28}(2), ASN^{H32}(4)	ALA^{475}[O]	THR^{H28}[N]	3.3
		ALA^{475}[O]	ASN^{H32}[ND2]	3.0
GLY^{476}	THR^{H28}(2)			
SER^{477}	THR^{H28}(1)			
**PHE^{486}**	VAL^{H2}(3), ARG^{H97}(2), ASP^{H105}(1)			
***ASN^{487}**	GLY^{H26}(2), PHE^{H27}(2), ARG^{H97}(3)	ASN^{487}[ND2]	GLY^{H26}[O]	2.9
		ASN^{487}[OD1]	ARG^{H97}[NH1]	3.6
		ASN^{487}[OD1]	ARG^{H97}[NH2]	3.2
***TYR^{489}**	ARG^{H97}(2), LEU^{H99}(5)	TYR^{489}[OH]	ARG^{H97}[NH1]	3.6
***GLN^{493}**	VAL^{H101}(1), TYR^{H102}(4)	GLN^{493}[NE2]	TYR^{H102}[OH]	2.7
		SER^{494}[O]	TYR^{L32}[OH]	3.7
TYR^{495}	TYR^{L32}(5)	TYR^{495}[O]	TYR^{L32}[OH]	2.9
		**GLY^{496}[O]**	SER^{L30}[OG]	3.3

Supplementary Table 2: Contacts between RBD and Fab2303.

Van der Waals contacts^{a}		Direct hydrogen bonds^{b}		
Spike	Fab 2212	Spike	Fab 2212	Distance (Å)
VAL^{445RBD1}	SER^{H74}(3)			
*ASN^{448RBD1}	THR^{H30}(5), THR^{H73}(1)	ASN^{448RBD1}[OD1]	THR^{H30}[OG1]	2.9
LEU^{455RBD1}	TYR^{H99}(1)			
PHE^{456RBD1}	TYR^{H100}(3)			
VAL^{483RBD1}	PRO^{L96}(5)			
*GLU^{484RBD1}	TYR^{H33}(5), TRP^{H50}(3), ASN^{H52}(1), PRO^{L96}(2)	GLU^{484RBD1}[OE2]	TYR^{H33}[OH]	2.5
		GLU^{484RBD1}[OE2]	ASN^{H52}[ND2]	3.4
GLY^{485RBD1}	TYR^{L90}(2)			
*PHE^{486RBD1}	TYR^{H106}(2), TYR^{L29}(1), LEU^{L31}(2), TYR^{L90}(6)	PHE^{486RBD1}[N]	TYR^{L90}[OH]	3.0
TYR^{489RBD1}	THR^{H98}(5), TYR^{H99}(4), TYR^{H100}(3)			
PHE^{490RBD1}	ASN^{H52}(1), SER^{H54}(4)			
*LEU^{492RBD1}	ASN^{H53}(3)	LEU^{492RBD1}[O]	ASN^{H53}[ND2]	3.5
*GLN^{493RBD1}	THR^{H30}(2), ASN^{H53}(3), THR^{H98}(1), TYR^{H99}(1)	GLN^{493RBD1}[NE2]	THR^{H30}[O]	3.6
		GLN^{493RBD1}[OE1]	THR^{H98}[OG1]	3.3
		GLN^{493RBD1}[OE1]	ASN^{H53}[ND2]	3.1
*SER^{494RBD1}	THR^{H30}(4), ASN^{H53}(2)	SER^{494RBD1}[N]	ASN^{H53}[OD1]	3.8
		SER^{494RBD1}[OG]	ASN^{H53}[OD1]	3.3
PHE^{342RBD2}	LEU^{H103}(1)			
VAL^{367RBD2}	TYR^{H100}(3)			
ASN^{370RBD2}	TYR^{H100}(3)			
*SER^{371RBD2}	TYR^{H100}(2), ASP^{H101}(3), ILE^{H102}(1)	SER^{371RBD2}[OG]	ILE^{H102}[N]	3.7
*ALA^{372RBD2}	TYR^{H99}(1), TYR^{H100}(2)	ALA^{372RBD2}[N]	TYR^{H100}[O]	3.2
*SER^{373RBD2}	ASP^{H101}(7)	SER^{373RBD2}[OG]	ASP^{H101}[OD1]	3.1
		SER^{373RBD2}[N]	ASP^{H101}[2D1]	3.8
PHE^{374RBD2}	ILE^{H102}(1), LEU^{H103}(1)			

Supplementary Table 3: Contacts between spike and TAU-2212.

a:Van der Waals contacts have interatomic distance ≤ 4.0 Å and defined by CCP4i.
b:Putative H-bonds and salt-bridges defined by PISA.
In the interface, RBD1 binds both with heavy and light chains. RBD2 only binds heavy chain.
H, heavy chain of mAb TAU-2212. L, light chain of mAb TAU-2212
*Stars indicate RBD residues, of which the side chain atoms are involved in forming hydrogen bonds with ACE2.
